# Immunostimulatory effects of Toll‐like receptor ligands as adjuvants in establishing a novel mouse model for pemphigus vulgaris

**DOI:** 10.1002/ctm2.1765

**Published:** 2024-07-19

**Authors:** Changxing Gao, Mei Liu, Yue Xin, Yong Zeng, Hui Yang, Xinyu Fan, Cheng Zhao, Bo Zhang, Lingzhi Zhang, Jing J. Li, Ming Zhao, Zijun Wang, Qianjin Lu

**Affiliations:** ^1^ Key Laboratory of Basic and Translational Research on Immune‐Mediated Skin Diseases Chinese Academy of Medical Sciences Jiangsu Key Laboratory of Molecular Biology for Skin Diseases and STIs Hospital for Skin Diseases, Institute of Dermatology, Chinese Academy of Medical Sciences and Peking Union Medical College Nanjing China; ^2^ Department of Dermatology The Second Xiangya Hospital of Central South University Changsha China; ^3^ Drum Tower Hospital Affiliated to Medical School of Nanjing University Nanjing China; ^4^ State Key Laboratory of Bioactive Substance and Function of Natural Medicines Beijing Key Laboratory of New Drug Mechanisms and Pharmacological Evaluation Study Department of Pharmacology Institute of Materia Medica, Chinese Academy of Medical Sciences and Peking Union Medical College Beijing China; ^5^ Laboratory of Molecular Immunology The Rockefeller University New York City New York USA

**Keywords:** adjuvant, animal model, pemphigus vulgaris, T cell response

## Abstract

**Background:**

The meticulous selection of appropriate vaccine adjuvants is crucial for optimizing immune responses. Traditionally, pemphigus vulgaris (PV), an autoimmune disorder, has been modelled using complete Freund's adjuvant (CFA). In this study, we aimed to discern potential variations in immune responses elicited by Toll‐like receptor (TLR) ligands as compared to CFA.

**Methods:**

A comprehensive investigation was conducted, comparing the effects of these adjuvants in conjunction with ovalbumin or desmoglein‐3. Flow cytometry was employed to analyse distinct cell subsets, while enzyme‐linked immunosorbent assay quantified antigen‐specific antibodies and cytokine levels. Histological examination of harvested skin tissues and transcriptome analysis of skin lesions were performed to identify differentially expressed genes.

**Results:**

TLR ligands demonstrated efficacy in inducing PV‐like symptoms in wild‐type mice, in contrast to CFA. This underscored the substantial impact of the adjuvant on self‐antigen tolerance. Furthermore, we proposed an enhanced method for establishing a PV model through adoptive transfer, substituting CFA with TLR ligands. Our results revealed that in contrast to the perception that CFA being the most potent immunopotentiator reported, CFA promoted regulatory T cells (Treg), follicular regulatory T cells and IL‐10‐producing neutrophils, whereas TLR ligands downregulated CCL17 and IL‐10. This suggested potential implications for the recruitment and activation of Treg subsets. While B cell and CD8^+^ T cell responses exhibited similarity, CFA induced less activation in dendritic cell subsets. A novel mouse model of PV and systemic comparison of immunostimulatory effects of adjuvants were provided by this study.

**Conclusions:**

The systematic comparison of CFA and TLR ligands shed light on the distinctive properties of these adjuvants, presenting innovative mouse models for the investigation of pemphigus. This study significantly contributes to adjuvant research and advances our understanding of PV pathogenesis.

**Key points/highlights:**

1. Immunization with desmoglein 3 and Toll‐like receptor (TLR) ligands effectively induces pemphigus symptoms in wild‐type mice, whereas complete Freund's adjuvant (CFA) fails.
2. TLR ligands heightened the autoreactivity of donor cells in the adoptive transfer pemphigus model.
3. CFA promoted regulatory T cells and IL‐10‐producing neutrophils, whereas TLR ligands downregulated CCL17 and IL‐10, leading to more effective immune responses.

## Graphical abstract

- Immunization with desmoglein 3 and Toll‐like receptor (TLR) ligands effectively induces pemphigus symptoms in wild‐type mice, whereas complete Freund's adjuvant (CFA) fails.
- TLR ligands heightened the autoreactivity of donor cells in the adoptive transfer pemphigus model.
- CFA promoted regulatory T cells and IL‐10‐producing neutrophils, whereas TLR ligands downregulated CCL17 and IL‐10, leading to more effective immune responses.

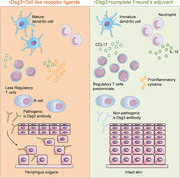

## 1 INTRODUCTION

The proper activation of innate immune cells plays a critical role in initiating adaptive immune responses. This holds true for both naturally occurring immune responses, such as those against infections and autoimmune diseases, as well as artificially induced responses, as seen in vaccination.^1^ Understanding the mechanisms by which the innate immune system facilitates the transition to adaptive immunity is a fundamental question in contemporary immunology research. Gaining a deeper understanding of this process will have profound implications for our comprehension of immune responses in the contexts of vaccination and autoimmunity, ultimately leading to improved strategies for immune intervention.

Vaccination stands as one of the most effective strategies for preventing and controlling infectious diseases. Adjuvants, which are commonly included in vaccines, serve to activate antigen‐presenting cells (APCs), particularly dendritic cells (DCs), which play a crucial role in shaping T cell differentiation, functionality and migration capacity. As a result, different adjuvants can lead to the generation of distinct T cell subsets, thereby influencing the outcome of immune responses. These responses encompass a range from breaking tolerance and inducing autoimmune reactions to eliciting protective immunity against specific pathogens in vaccine‐induced settings. While numerous studies have investigated the properties of vaccine adjuvants in stimulating immune responses against foreign antigens,^2^, ^3^, ^4^ their relevance to self‐antigens remains unclear. Investigating this aspect could be highly valuable in establishing animal models of autoimmune diseases induced by immunization. Currently, animal models of autoimmune diseases often depend on the use of well‐characterized autoantigens.^5^, ^6^, ^7^ A better understanding of the applicability of vaccine adjuvants in self‐antigen settings would provide insights into the underlying mechanisms of these diseases and facilitate the development of more effective therapeutic strategies. By bridging this knowledge gap, we can gain valuable insights into the interplay between adjuvants and self‐antigens, advancing our understanding of autoimmune diseases and their experimental models.

Pemphigus vulgaris (PV) is an autoimmune bullous skin disease characterized by the presence of autoantibodies targeting desmoglein‐3 (Dsg3), which is a cell−cell adhesion molecule found in desmosomes.^8^, ^9^, ^10^ The binding of pathogenic antibodies to Dsg3 induces a loss of adhesion between keratinocyte in the epidermis, leading to blister formation, painful erosions and potentially fatal complications. Animal models play a crucial role in the development of safe and effective therapies for PV. However, the availability of adult PV animal models is limited, with the adoptive transfer mouse model being the most widely used.^10^, ^11^, ^12^, ^13^, ^14^ This model requires both *Dsg3* knock‐out mice as donor mice and immunodeficient mice as recipient mice. Despite its utility, the adoptive transfer mouse model has certain limitations. Firstly, it necessitates the use of two distinct gene knockout mouse strains, which increases cost and complexity. Secondly, it fails to mimic the process of self‐tolerance loss to Dsg3 since the recipient mice lack self‐tolerance. Thirdly, the immune responses of Dsg3‐reactive donor cells may not be regulated by pre‐established immune tolerance, thus limiting its applicability for investigating immunotherapies based on regulatory cells. Therefore, a clear need exists for novel PV animal models that exhibit overactivated yet controllable immune responses, addressing these limitations and providing a more comprehensive understanding of PV pathogenesis and the development of therapeutic interventions. Previous studies demonstrated that antibodies induced by immunization with rDsg3 in wild‐type mice, constrained by immune tolerance, failed to produce pathogenic antibodies capable of recognizing native Dsg3.^15^, ^16^, ^17^, ^18^ Notably, the adjuvants used in these studies were complete Freund's adjuvant (CFA), alum,^15^, ^17^, ^19^ squalene‐based TiterMax^18^ or unspecified.^11^ Although CFA based on mycobacteria remains the most potent immunopotentiator reported to date,^20^ we believe that the recent availability of vaccine adjuvants with excellent immune‐activation capabilities opens up possibilities for inducing Dsg3‐specific autoimmune responses.

Extensive research has been conducted on the modulation of immune response by the selection of appropriate vaccine adjuvants.^21^, ^22^, ^23^ For instance, the use of a triple combination of three Toll‐like receptor (TLR) ligands (TLR2/6, TLR3 and TLR9) has been shown to enhance the protective efficacy of HIV envelope peptide vaccination in mice.^24^ This combination increases the production of DC IL‐15, along with its receptor IL‐15Rα, ultimately boosting the functional antigen avidity. Berberine, a natural product, has also demonstrated its potential as an adjuvant for novel vaccines. It has been found to improve the formation of central memory cells (Tcm) while reducing effector proliferation. This effect is achieved through the activation of the AMPK (AMP‐activated protein kinase) and Stat5 pathways.^25^ In the context of autoimmune disease treatment, the induction or expansion of regulatory T cells (Treg) holds significant importance. Several substances have been identified as potential suppressants capable of stimulating regulatory T cell responses. These include CDK8/19 inhibitor,^26^ a tryptophan metabolite,^27^ alum^28^, ^29^ and cytokines.^30^ These substances hold promise as candidates for the development of therapeutic vaccines in autoimmune diseases.

In this study, our primary aim was to determine whether immunization with rDsg3 and an alternative adjuvant candidate, instead of CFA, could induce a phenotype resembling PV in wild‐type mice. We successfully developed a novel, active PV mouse model by immunizing wild‐type C57BL/6J mice with the adjuvanted rDsg3 protein. This model closely mimicked the clinical symptoms observed in patients with the mucocutaneous type of PV. Transcriptome analysis of skin lesions from the PV mouse model revealed the downregulation of CCL17 after treatment with TLR ligands. Furthermore, we demonstrated the enhanced efficacy of TLR ligands in an adoptive transfer PV model. Lastly, we utilized the model antigen ovalbumin (OVA) to investigate the immune response priming by both TLR ligands and CFA, serving as a comparative approach to assess their immunological properties.

## 2 RESULTS

### 2.1 Immunization with rDsg3 and active adjuvants induced pemphigus‐like symptoms in wild‐type mice

**FIGURE 1:**
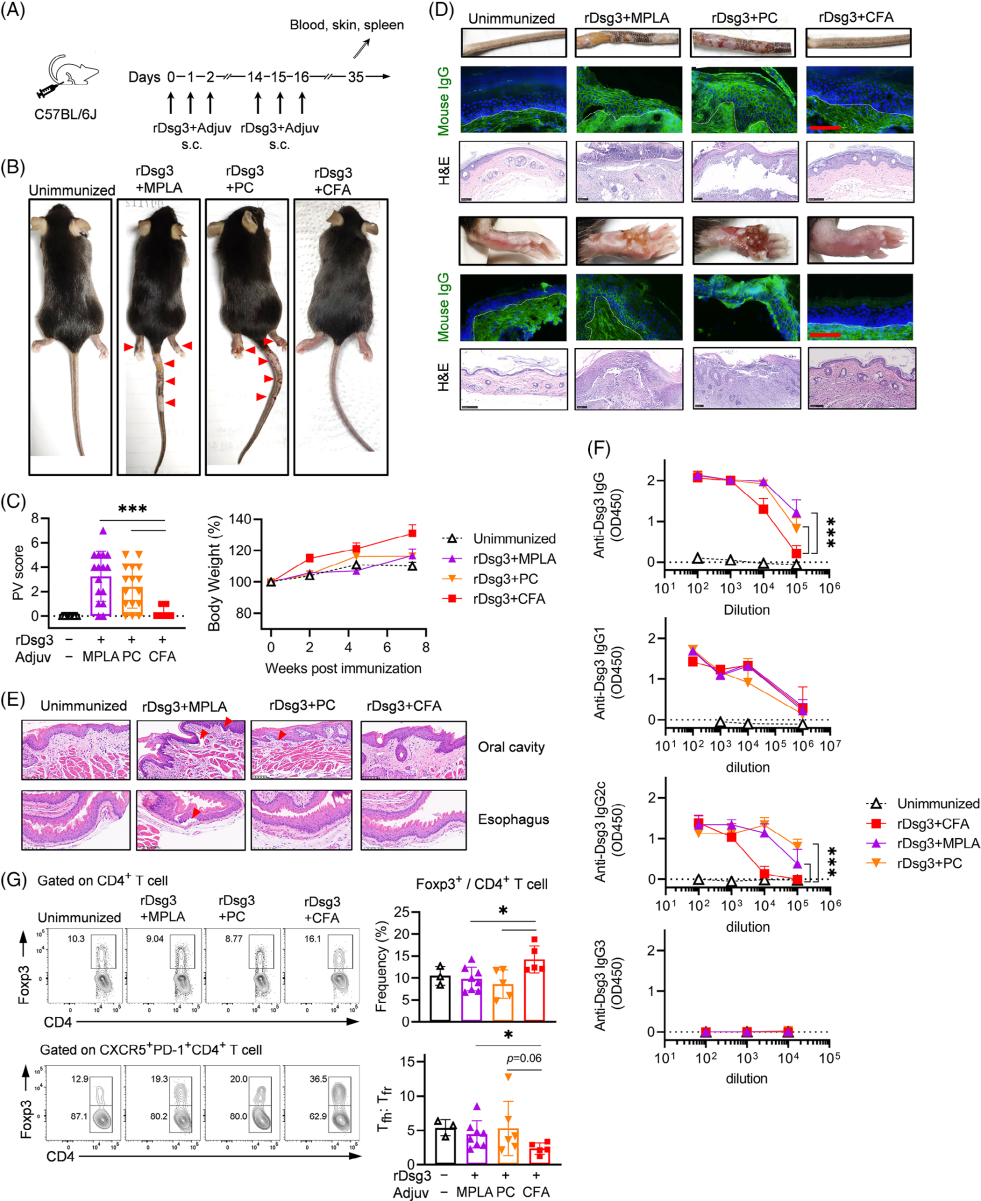
Direct immunization induced PV symptoms in wild‐type mice. (a) A schematic depicting the treatment of mice is shown. Mice were subjected to a prime‐boost regime with rDsg3 and various adjuvants as indicated. After 35 days from the first immunization, plasma or serum was obtained from peripheral blood, and skin or mucosal tissues were collected for histological analysis, lymphocytes were harvested from spleen for flow cytometry analysis. (b) The gross phenotype of PV model mice. Red arrowhead indicates sites with skin erosion. (c) Changes in body weight were recorded bi‐weekly, and PV scores were assessed 28 days after the initial immunization. The PV score data were pooled from five experiments. (d) Skin tissues from the tail and footpad were harvested for H&E staining or direct immunofluorescence (IF). The borders of epidermis and dermis were indicated with white dash lines. (e) Histological examination was performed on mucosal tissues from the oral cavity and oesophagus. Red arrowhead indicates sites of acantholysis. Scale bar: 100 μm. (f) Dsg3‐specific IgG, IgG1, IgG2c and IgG3 were measured by ELISA. OD values at serial dilution read at 450 nm are shown. (g) Flow cytometry analysis of Foxp3^+^ CD4^+^ Treg, ratio of Tfh and Tfr cells in spleen: CD8^–^CD4^+^ T cells and CD8^–^CD4^+^CXCR5^+^PD‐1^+^ T cells were gated for the analysis, respectively. The results presented are from three to four independent experiments with similar outcomes and are shown as mean ± SD. Statistical significance is indicated by *p < .05 and ***p < .001 denoting a significant difference between groups. A common symbol was assigned above the two connection lines if they were of the same significance. Adjuv, adjuvant; CFA, complete Freund's adjuvant; MPLA, monophosphoryl lipid A; ns, not significant; PC, poly(I:C)+CpG 1826.

After constructing and verifying recombinant mouse Dsg3 (Figure S1), we aimed to investigate whether combining rDsg3 with active adjuvants could directly induce a PV phenotype in wild‐type C57BL/6 mice (Figure 1a). CFA (mixed mechanism including NOD‐like receptor (NLR), inflammasome, Mincle agonist), monophosphoryl lipid A (MPLA, TLR4 ligand, derived from lipopolysaccharide or LPS) or combined use of polyinosinic‐polycytidylic acid (poly(I:C), TLR3 ligand, model of viral dsRNA) and unmethylated CpG motif–based oligodeoxynucleotide (CpG 1826, TLR9 ligand, model of bacterial DNA) were elected as adjuvants in this study. We observed that immunization with rDsg3 adjuvanted with a single or combination of TLR ligands induced severe skin erosion in the footpad and tail of wild‐type mice 14 days after boost (Figure 1b). Further analysis of PV symptoms revealed that TLR ligands induced a significantly higher PV score compared to CFA (*p *< .001; Figure 1c). The immunization with TLR ligands resulted in a PV phenotype with circumscribed involvement of the tail and foot (Table 1) and a relatively lower proportion of alopecia compared to the classic adoptive transfer PV model.^12^, ^13^ Histological examination using hematoxylin and eosin (H&E) staining demonstrated suprabasilar blistering with acantholysis in the skin tissues, while direct immunofluorescence (IF) showed IgG deposition between keratinocyte in the epidermis of PV model mice, confirming the successful induction of PV (Figure 1d). Although no noticeable body weight loss was observed (Figure 1c), acantholysis in the oesophageal and oral mucosal epithelium was detected in 50−60% of the model mice (Figure 1e and Figure S2b). The skin‐dominant PV phenotype may be attributed to skin‐topical T cells migrating from the draining lymph node (LN) after subcutaneous immunization, as DCs from the skin‐draining LNs can impart skin‐homing properties on T cells during priming.^31^, ^32^ Enzyme‐linked immunosorbent assay (ELISA) measurement of anti‐Dsg3 antibodies in plasma collected at day 35 demonstrated a significant increase in PV model mice compared to the unimmunized and CFA groups (*p *< .001; Figure 1f). Further characterization of the IgG subclass showed that IgG1 predominated in plasma, which was consistent with previous report.^33^ While IgG1 level was comparable in immunized mice, IgG3 was not detected, indicating the significant difference in anti‐Dsg3 antibodies might be attributed to IgG2c (*p *< .001; Figure 1f). To characterize the effect of the adjuvant alone on loss of tolerance, we tested the adjuvants by themselves and found they were unable to induce PV phenotype and anti‐Dsg3 antibodies (Figure S2c,d), indicating the vital role of the presence of the self‐antigen. Adjuvant‐activated DC might have no access to the desmoglein, which is a self‐structure protein.

**TABLE 1:** Summary of pemphigus vulgaris score and the incidence of phenotypic symptoms in the immunization‐induced PV model.

Type of lesion	Sites	Score	Incidence (%)			
			Unimmunized	rDsg3+MPLA	rDsg3+PC	rDsg3+CFA
Erosion	muzzle	1	0	0	0	0
	periocular	1	0	19	6	0
	periauricular	1	0	6	0	0
	back	1	0	13	0	0
	chest	1	0	0	0	0
	abdomen	1	0	6	0	0
	right foreleg	1	0	0	0	0
	left foreleg	1	0	0	6	0
	right hind leg	1	0	69	44	0
	left hind leg	1	0	63	50	0
	tail	1−2^a^	0	38	56	0
Alopecia	face	1	0	13	6	0
	neck	.5	0	6	0	0
	back	1	0	19	6	0
	abdomen	.5	0	25	6	0
Erythema	footpad	1	0	38	31	31
	abdomen	1	0	0	0	0

Abbreviation: PC, poly(I:C)+CpG 1826.

^a^ The scoring system included assigning a score of 1 to mice with scattered skin erosion sites and a score of 2 to those with a large continuous lesion area in the tail.

Furthermore, we found that, despite the presence of anti‐Dsg3 antibodies, CFA failed to facilitate the breach of tolerance against self‐Dsg3, as evidenced by the lack of significant gross phenotype changes manifested by paw swelling and slight erythema and histological examination findings. To evaluate the pathogenicity of serum from model mice, a passive neonatal PV model was used. We were not surprised to find all of the plasma or sera from mice immunized with TLR ligands was pathogenic in neonatal mice (Figure S2), in contrast, none of the tested plasma from mice immunized with CFA produced IgG deposition between keratinocytes, as revealed by direct IF. However, some plasma induced moderate loss of keratinocyte attachment, as examined by H&E staining (Figure S2), indicating that the pathogenicity of anti‐Dsg3 antibodies induced by CFA was still restrained by immune tolerance. The failure of breach of tolerance might be attributed by the increased proportion of Foxp3^+^CD4^+^ T cell (*p *< .05) and lower ratio of follicular helper cells (Tfh) to follicular regulatory T cells (Tfr) (Figure 1g) in the spleen of mice immunized with CFA.

### 2.2 Active adjuvants enhanced the autoreactivity of *Dsg3^–/–^* donor cells in the adoptive transfer PV model

**FIGURE 2:**
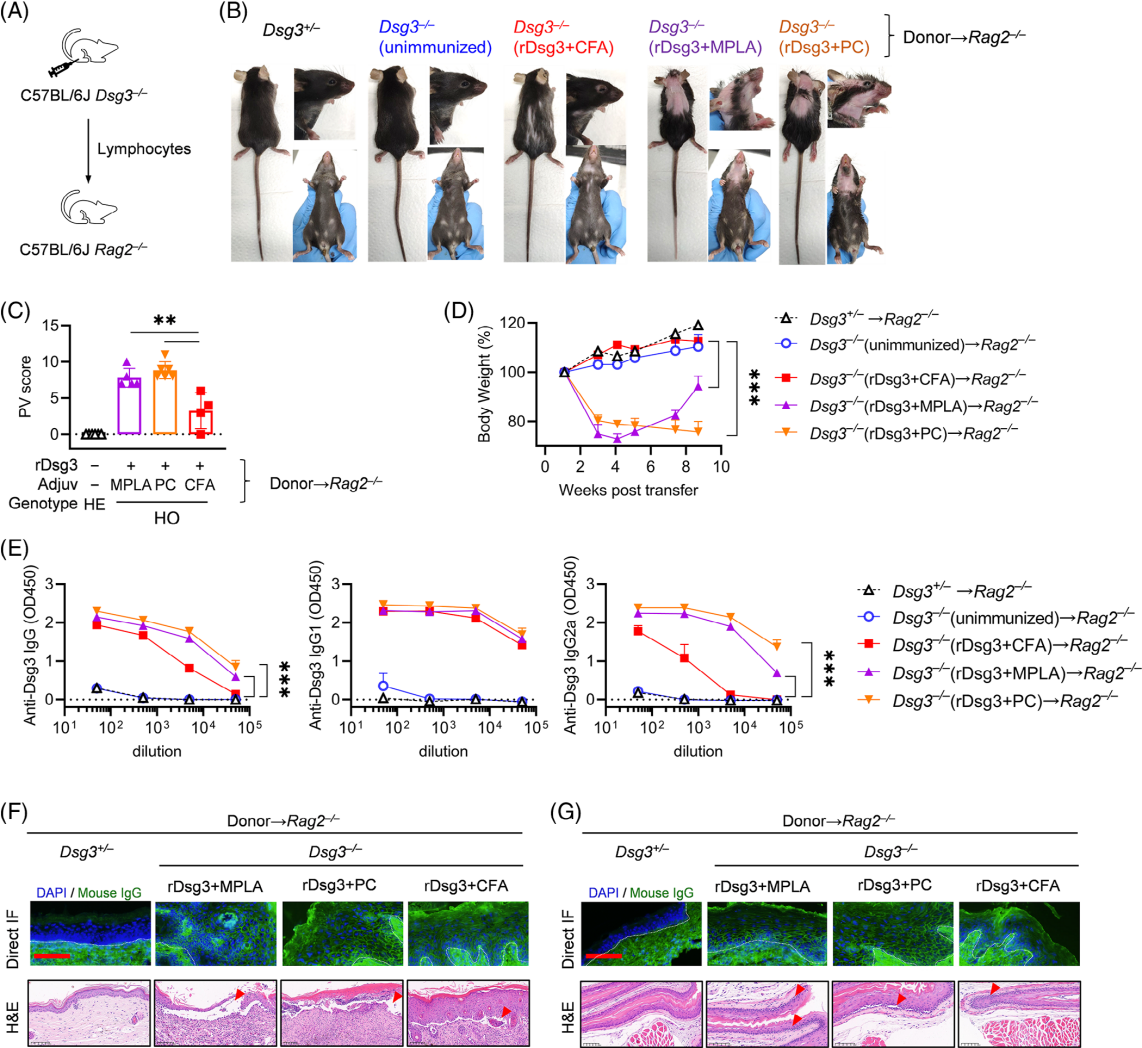
Optimization of adoptive transfer PV model using TLR ligands. (a) Experimental setup: *Dsg3^–/–^* mice were immunized with rDsg3 and different adjuvants in a prime‐boost regime. After 35 or 49 days, lymphocytes were isolated from the spleen and injected into *Rag2^–/–^* mice via the tail vein. Body weight and phenotype were monitored at specified time points. (b) Gross phenotype of mice: Alopecia and skin erosion phenotype observed in mice. (c) PV score assessment at 28 or 40 days after transfer. PV score data were pooled from two experiments. (d) Weekly changes in body weight normalized to initial body weight. (e) Measurement of Dsg3 specific IgG, IgG1 and IgG2c in plasma or serum obtained at day 40 by ELISA. OD values at 450 nm are shown. Skin (f) and mucosal tissues (g) were harvested for H&E staining or direct immunofluorescence. The borders of epidermis and dermis were indicated with white dash lines. Scale bar: 100 μm. Representative results from two independent experiments are presented as mean ± SD. *p < .05 and ***p < .001 indicate a significant difference between groups. HE, hemizygote; HO, homozygote.

Considering the characteristics of the tested active adjuvants in priming autoimmune responses, we asked whether TLR ligands could be used in the classic adoptive transfer PV model to facilitate the establishment of Dsg3‐reactive immune responses. After constructing and verifying *Dsg3^–/–^* mice at the gene and protein level (Figure S1a,b), these mice were immunized with rDsg3 and active adjuvants in a prime‐boost regimen at day 0−2 and day 14−16. Lymphocytes were isolated 1−2 weeks after the boost immunization and transferred to *Rag2^–/–^* mice, which had been confirmed to lack T cell and B cell by flow cytometry (Figure 2a and Figure S1e). While donor cells primed with CFA induced a slightly alopecia phenotype, mice receiving donor cells from TLR‐primed mice exhibited the typical phenotype of PV, including balding and erosion on the ear, cheek, snout, footpad and tail (Figure 2b), along with a higher PV score than CFA (*p *< .01; Figure 2c). Moreover, gross observations revealed that mice receiving donor cells from *Dsg3^+/–^* or unimmunized or CFA‐primed *Dsg3^–/–^* mice showed almost no body weight loss, compared to those immunized with TLR and rDsg3. The latter group exhibited severe body weight loss, with some mice reducing their body weight to 70% at 4 weeks post‐transfer (*p *< .001; Figure 2d). Serologically, donor cells primed with TLR ligands produced a higher level of anti‐Dsg3 IgG and IgG2c (*p *< .001; Figure 2e). Histologically, skin and mucosal tissues were harvested 40 days after transfer, H&E staining demonstrated suprabasilar blistering with acantholysis in the skin (Figure 2f) and mucosal tissues (Figure 2g). Direct IF revealed IgG deposition between keratinocyte in the epidermis of PV model mice, confirming the PV phenotype. To optimize the experimental setup, we initially transferred varying numbers of donor cells to determine the most effective method. As expected, we found that a higher number of donor cells primed with CFA was necessary to induce PV (Figure S3). These findings suggest that the use of CFA could be replaced with TLR ligands, providing an improved method for establishing the adoptive transfer of PV.

### 2.3 TLR ligand suppressed CCL17 expression and reduced Treg recruitment

**FIGURE 3:**
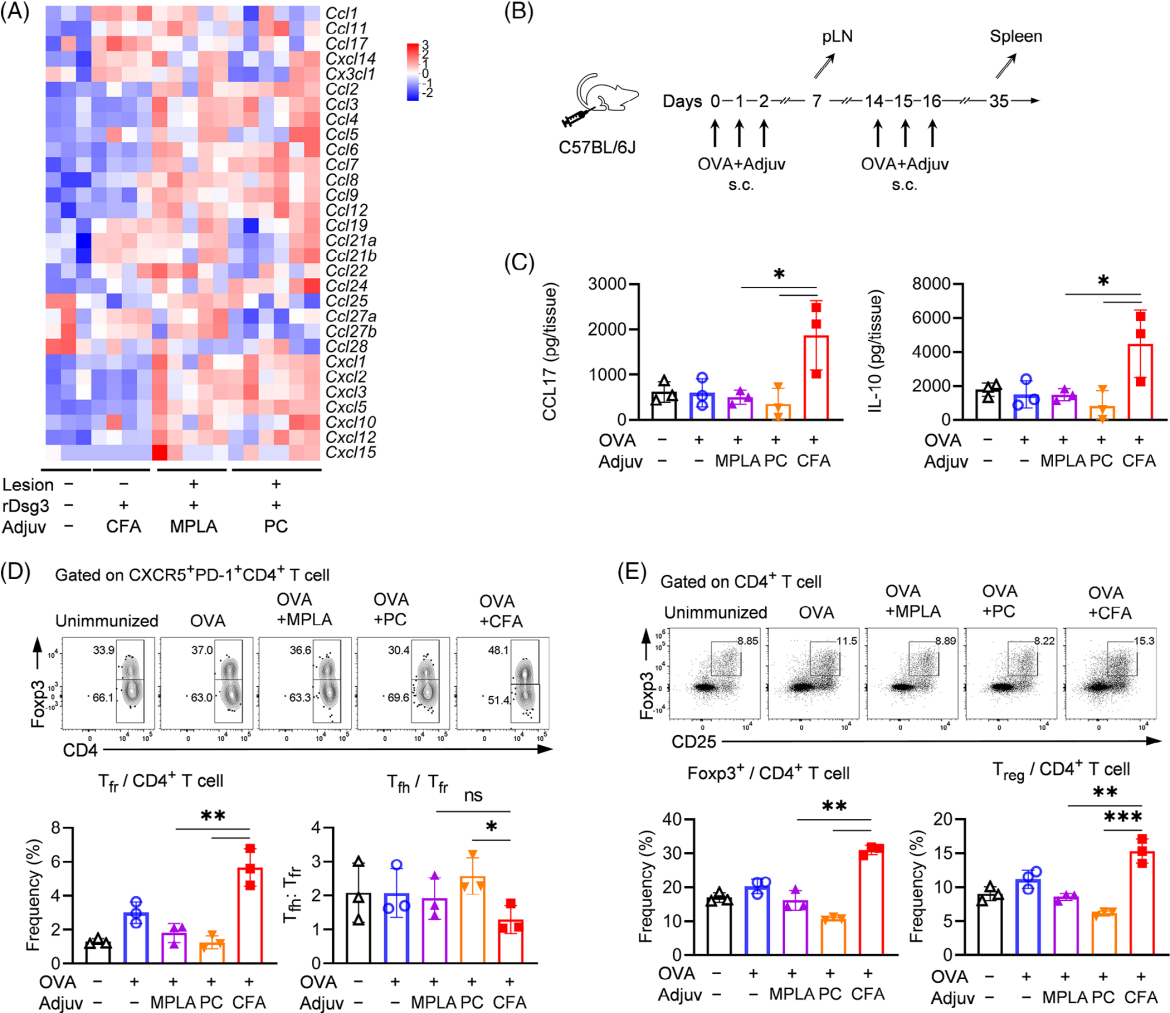
CCL17 downregulation by TLR ligands correlates with impaired regulatory CD4^+^ T cell responses. (a) RNA‐seq analysis: Skin lesions from the immunization‐induced PV model mice (as shown in Figure 1b) were harvested and homogenized for RNA‐seq. Heatmap visualization of chemokines and integrins transcriptome analysis. (b) Mice were immunized with OVA and different adjuvants in a prime‐boost regime. Spleens were harvested for flow cytometry analysis 35 days after the first immunization. (c) Cytokine quantification: Lymph nodes were homogenized for quantification of cytokines by ELISA. (d) Flow cytometry analysis of Tfh and Tfr cells in spleen: CD8^–^CD4^+^CXCR5^+^PD‐1^+^ T cells were gated for the analysis of the proportion of Tfh and Tfr. (e) Flow cytometry analysis of Foxp3^+^ CD4^+^ Tregs in the spleen gated on CD8^–^CD4^+^ T cells. The results are representative of two independent experiments and presented as mean ± SD. *p < .05, **p < .01 and ***p < .001 indicate a significant difference between groups.

To further investigate the underlying mechanisms of TLR ligands and CFA in autoimmunity, skin lesions from mice in the immunization‐induced PV model were harvested at day 40 to measure the mRNA levels of chemokines. In mice immunized with rDsg3 and TLR ligands, most chemokines were upregulated, indicating enhanced recruitment of proinflammatory cells. However, CCL17 expression was upregulated after CFA treatment, while TLR ligands showed a downregulatory effect (Figure 3a). To better understand the differences between CFA and TLR ligands in response to foreign antigens, we utilized OVA as a model foreign antigen and performed prime‐boost immunization (Figure 3b), harvest the LNs at d7 and spleens at d35 for analysis. The increased CCL17 expression was further confirmed in the LN homogenates by ELISA at the protein level (Figure 3c). CCL17 has been shown to specifically attract CCR4^+^ Tregs, and blocking Treg migration with a CCR4 antagonist has the potential in promoting anti‐tumour immunity.^34^, ^35^ We hypothesized that increased CCL17 levels might stimulate the accumulation of Tregs in the draining LNs, thereby limiting the activation and magnitude of the T cell response.

During the priming phase at day 7, CFA induced a high number of CD4^+^ T cells (Figure S4a), but the proportion of follicular helper cells (Tfh) was relatively low (Figure S4b), with a high level of follicular regulatory T cells (Tfr) and a lower Tfh:Tfr ratio (*p *< .05; Figure S4b,c). This suggests that the full induction of T cell magnificence was not achieved. The total Foxp3^+^CD4^+^ T cell was also significantly increased in mice receiving OVA+CFA (*p *< .05; Figure S4d). Next, we examined the secondary CD4^+^ T cell response after boost immunization at day 35. While the proportions of Tfh cells were comparable among different adjuvants, CFA induced a higher proportion of total Tregs and Tfr cells in the secondary response after repeated exposure to CFA compared to TLR ligands (*p *< .05, *p *< .01; Figure 3d,e and Figure S4b). These results were quite consistent with those obtained when immunized with Dsg3 (Figure 1g).

**FIGURE 4:**
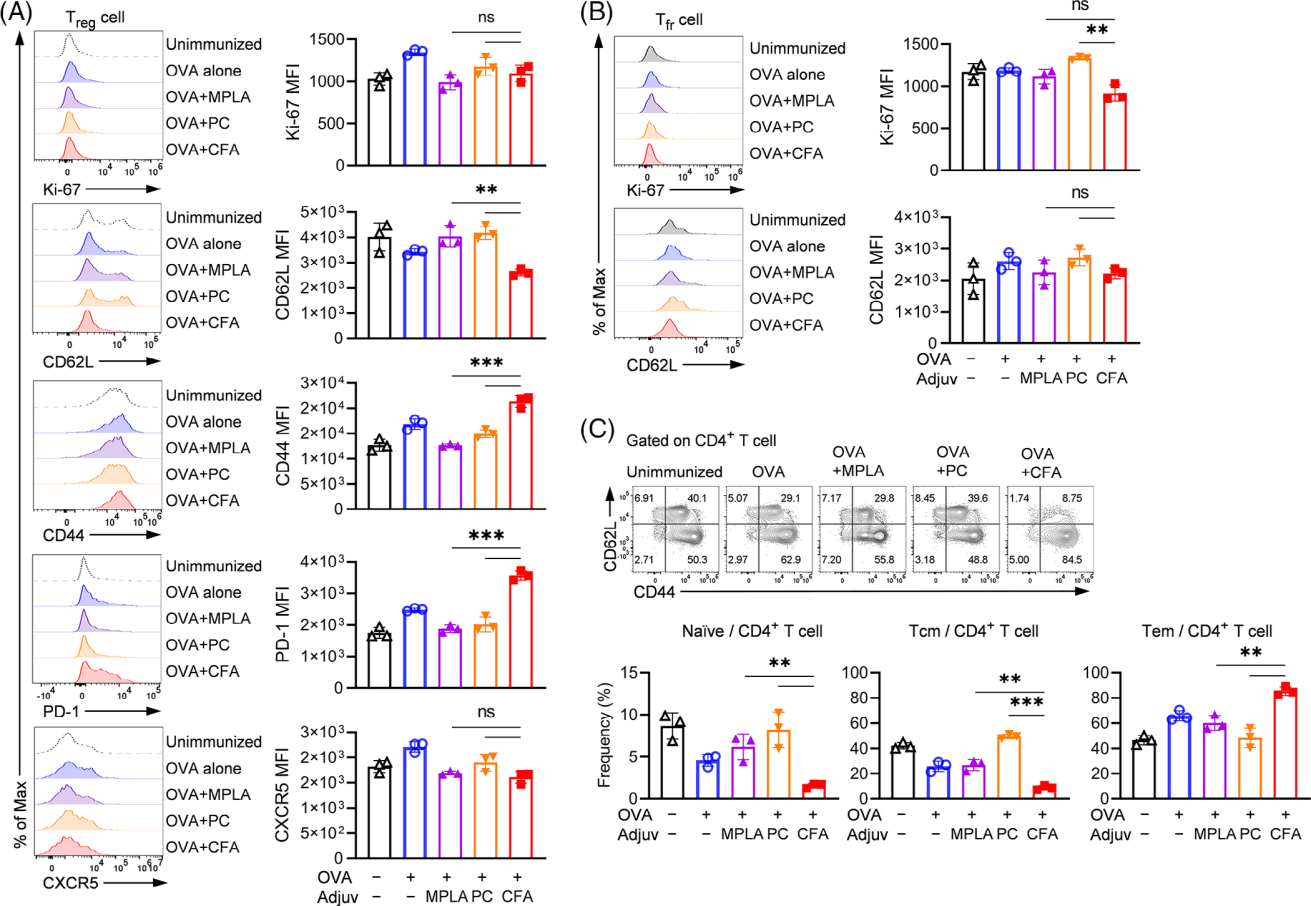
Induction of regulatory T cell with a less‐activated phenotype by TLR ligands. Tregs (a) and Tfr (b) in the spleen were gated for analysis of the expression of activation markers. (c) Flow cytometry analysis of CD4^+^ T cell phenotyping. The results are representative of two independent experiments and shown as mean ± SD. *p < .05, **p < .01 and ***p < .001 indicate a significant difference between groups. MFI, mean fluorescence intensity; Tcm, central memory T cell; Tem, effector memory T cell.

Upon examining the activation and proliferation capacity of Tregs in the spleen, we observed no improvement in the expression of Ki‐67 in Tregs from mice immunized with CFA (Figure 4a and Figure S5b). Interestingly, the mean fluorescence intensity (MFI) of Ki‐67 in Tfr was lower compared to poly(I:C)+CpG 1826 (refered to as PC) (*p *< .001; Figure 4b), suggesting that the increased Tregs might result from elevated recruitment guided by CCL17 rather than local T cell proliferation. CFA also increased the expression of CD44 and PD‐1 and downregulated CD62L in Treg cells, indicating a high activation status (*p *< .01 or .001; Figure 4a). The Tfr cells also exhibited lower CD62L expression, although not significantly (Figure 4b). Furthermore, we found that CFA+OVA induced a highly activated CD4^+^ T cell population, with effector memory cells (Tem) predominating and a lower proportion of central memory cells (Tcm) and naïve cells (Figure 4c). To better understand the effect of CFA on antigen‐specific CD4^+^ T cell, splenocytes from OVA‐immunized mice were stimulated with antigenic epitope (ova_323‐339_) to upregulate OVA‐specific T cell receptor (TCR) and incubated with class II tetramer loaded with ova_329‐337_ (Figure S6a,b). OVA‐specific CD4^+^ T cells were detected by tetramer staining and we found the frequencies of Treg and Tfr in tet^+^ CD4^+^ T cell were significantly higher in the CFA group compared to TLR ligands (Figure S6c). Although no difference was observed in memory phenotype, the proportion of Treg in CD44^+^CD62L^–^ population increased with CFA immunization (Figure S6c). These findings suggest that CFA promoted activation of antigen‐specific regulatory T cell.

Additionally, the activation and proliferation markers of Treg in the spleen from PV mice induced by immunization were examined, showing no difference between CFA and TLR ligands (Figure S5b,c). This implies that both CFA and TLR ligands equally contribute to the activation and proliferation of Treg and Tfr during immunization with self‐antigen. We also found that CFA+rDsg3 had a distinct effect on memory cell subsets, reducing Tem and increasing naïve cells (Figure S5d). Intracellular cytokine staining revealed that TLR ligands induced higher production of IFN‐γ and similar level of TNF‐α, IL‐2 in CD4^+^ T cells (Figure S5e). In summary, TLR ligands reduced the recruitment of Tregs compared to CFA, possibly due to downregulated CCL17 levels.

### 2.4 Comparable antigen‐specific CD8^+^ T cell and B cell responses were elicited by TLR ligand and CFA

To comprehensively assess the immune responses induced by TLR ligands and other adjuvants, we further investigated the responses of CD8^+^ T cells and B cells.

**FIGURE 5:**
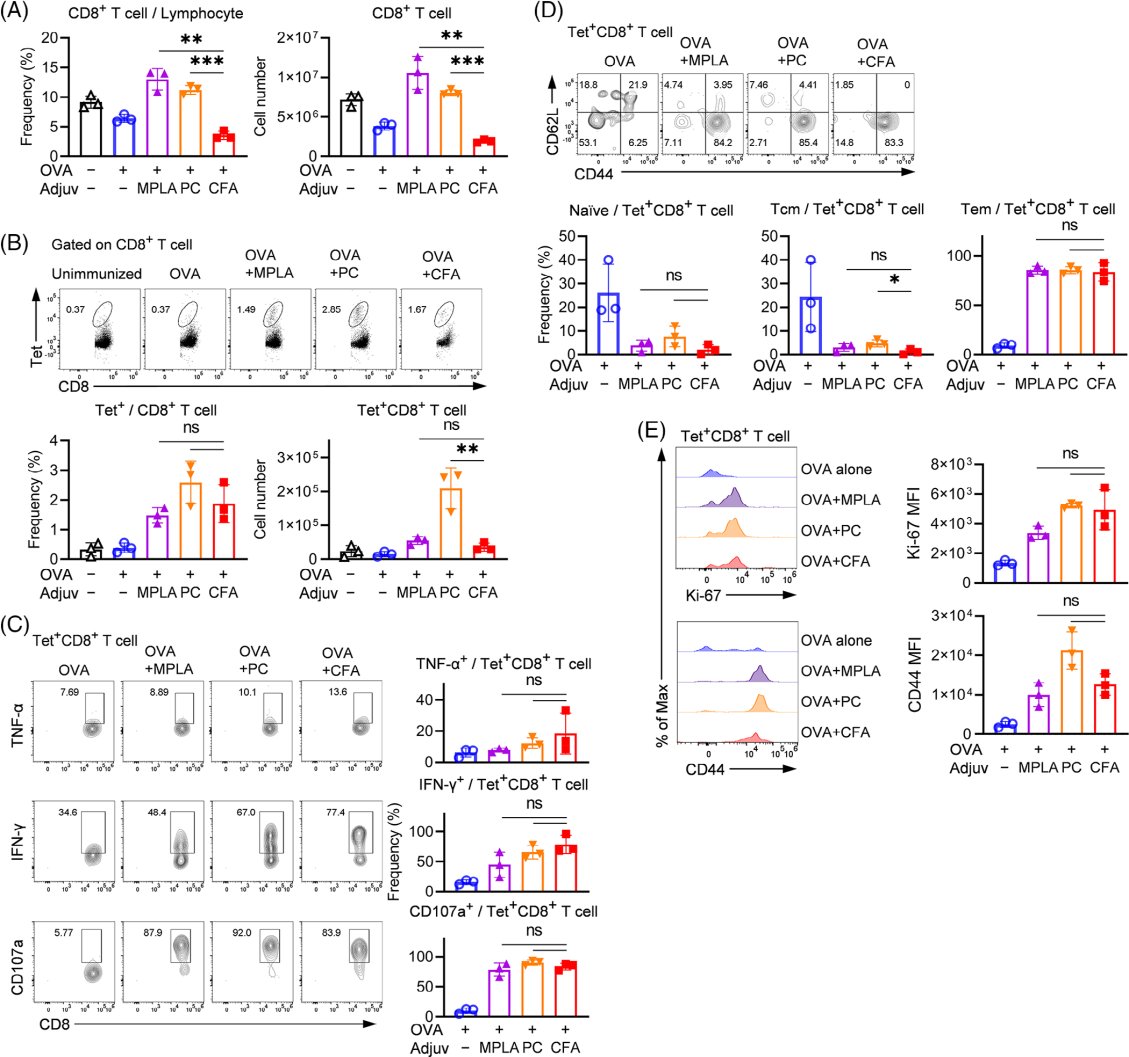
Enhanced CD8^+^ T cell responses by TLR ligands and CFA. Mice were immunized with OVA and various adjuvants following a prime‐boost regime (same as in Figure 3b). Spleen samples were collected 35 days after the prime immunization. (a,b), The frequency and cell number of total CD8^+^ T cells (a) and antigen‐specific CD8^+^ T cells (b) were analysed by flow cytometry. (c) Flow cytometry analysis of Tet^+^CD8^+^ T cell functionality by CD107a and intracellular cytokine staining. (d) The memory phenotype of Tet^+^CD8^+^ T cells was determined based on the expression of CD44 and CD62L. (e) Tet^+^CD8^+^ T cells were gated for the analysis of the proliferation capacity and activation status of antigen‐specific CD8^+^ T cells by examining the expression of Ki‐67 and CD44. The results are representative of two independent experiments and are shown as mean ± SD. Statistical significance between groups is indicated by *p < .05, **p < .01 and ***p < .001. Tet represents MHC‐I tetramer loaded with SIINFEKL.

CFA induced a larger number of CD8^+^ T cell in the draining LN (Figure S7a) but the number was less than TLR ligands in the spleen (Figure 5a). Although PC induced a higher number of antigen‐specific tet^+^ CD8^+^ T cells compared to CFA (*p *< .01; Figure 5b) after boost immunization, the proliferation capability, activation status and cytotoxicity phenotype were similar among all the adjuvants tested, as indicated by positive cells for IFN‐γ, CD107a and TNF‐α (Figure 5c) and the expression of Ki‐67, CD44 (Figure 5e and Figure S7d). Although more antigen‐specific CD8^+^ T cell expressing CD107a and TNF‐α were induced by PC compared with CFA during the priming phase in the LN (Figure S7c), there were no differences among adjuvants in the spleen after boost immunization. The memory cell subset composition of antigen‐specific CD8^+^ T cells exhibited a similar pattern across different adjuvants, with Tem cells being predominant and a small proportion of Tcm and naïve cells. In contrast, approximately 20% of cells induced by OVA alone remained in a naïve state (Figure 5d). We also examined the total CD8^+^ T cell responses in the immunization‐induced PV model and found no difference among adjuvants tested (Figure S8a,b). Based on these findings, we conclude that CFA did not have any distinct effect on CD8^+^ T cell responses.

**FIGURE 6:**
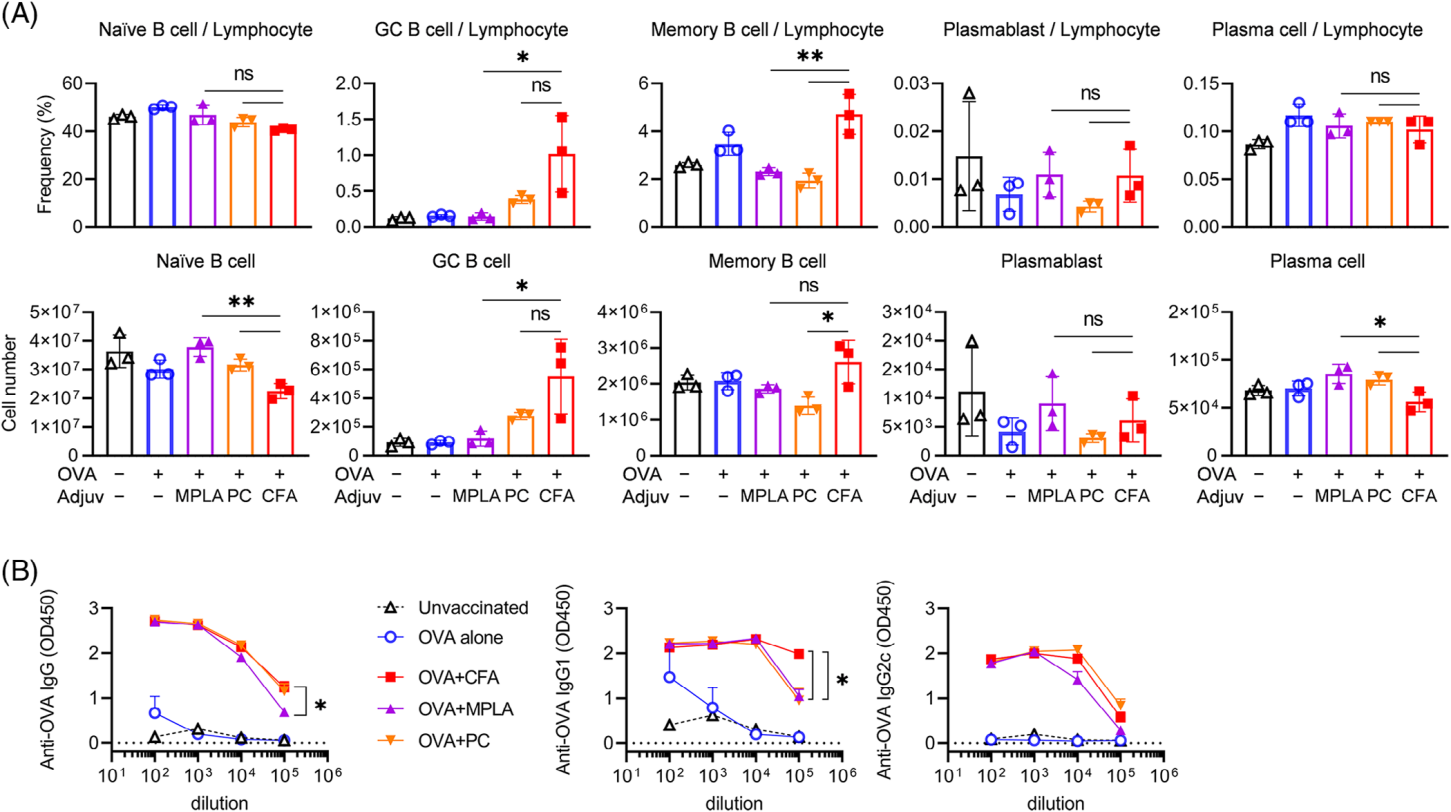
Comparable humoral responses elicited by TLR ligands and CFA. Mice were immunized with OVA and different adjuvants as indicated following a prime‐boost regime. Spleens were collected for flow cytometry 35 days after initial immunization. Plasma or serum samples were obtained from peripheral blood at day 35. (a) The proportion and absolute number of B cell subsets in the spleen were analysed by flow cytometry, as illustrated in Figure S9. CD8^–^CD4^–^CD19^+^ or B220^+^ B cells were gated for analysis. (b) OVA‐specific IgG, IgG1 and IgG2c were measured by ELISA. OD values at serial dilution were read at 450 nm. The results are representative of two independent experiments and shown as mean ± SD. Statistical significance between groups is indicated by *p < .05, **p < .01 and ***p < .001. GC denotes germinal centre, and ns represents not significant.

Furthermore, we determined the proportions of B cell subsets using flow cytometry (the gating strategy is shown in Figure S9a). We found that comparable frequencies of naïve B cells and plasma cells were generated in both the peripheral lymph nodes (pLNs) and spleen. However, we observed that the CFA elicited amplified germinal centre responses specifically in the spleen (Figure 6a and Figure S9b). The frequencies of memory B cells and germinal centre B cells were higher with CFA compared to TLR ligands (Figure 6a and Figure S9b). However, these differences were not observed in the immunization‐induced PV model, where CFA induced a higher proportion of plasma cell (Figure S8c). This inconsistency may arise from the unique properties of the antigen and the diverse CD4^+^ T cell responses involved. Additionally, ELISA was performed to measure the quantities of anti‐OVA IgG. The optical density (OD) measurements of CFA and TLR ligands exhibited a similar decreasing trend as the plasma dilution increased. Notably, these OD values were consistently higher than those obtained from mice treated with OVA alone or from naïve mice (Figure 6b). These results suggest that the administration of CFA has the potential to elicit humoral responses against foreign antigens that are not only comparable but, in some cases, even surpass those produced by other means.

### 2.5 Adjuvants stimulated distinct functions in APCs and IL‐10 producing neutrophils

**FIGURE 7:**
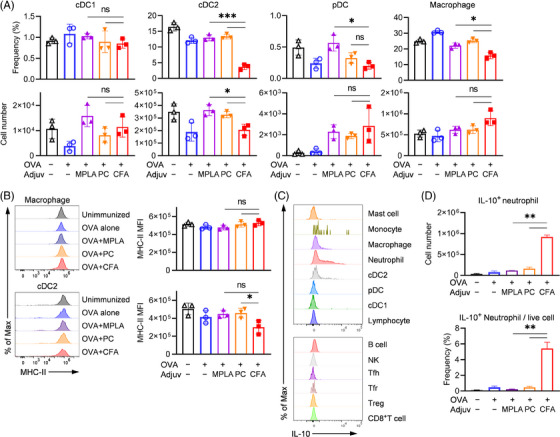
Incomplete activation of dendritic cells (DCs) by CFA. The gating strategy for analysing the DC subset and macrophages by flow cytometry is shown in Figure S10. (a) The cell number and frequency of APCs in spleen were analysed. (b) The presentation functionality of cDC2 and macrophage in spleen were characterized by the expression of MHC‐II. (c) Cell populations were overlapped in histogram for comparison of the capacity of IL‐10 production of major innate and adaptive immune cells. (d) Flow cytometry analysis of the cell number and frequency of IL10^+^ neutrophils in the spleen. The results are representative of two independent experiments and shown as mean ± SD. Statistical significance between groups is indicated by *p < .05, **p < .01 and ***p < .001. DC, dendritic cell; cDC1, conventional DC 1; cDC2, conventional DC 2; MHC‐II, major histocompatibility complex class II molecule; pDC, plasmacytoid DC.

We examined the presence of various APCs subsets in secondary lymphoid organs. These subsets included conventional dendritic cells 1(cDC1), cDC2, plasmacytoid dendritic cells (pDCs) and macrophages (Figure S10a). We observed that TLR ligands led to an increased accumulation of DCs and macrophages in the pLN (*p *< .05 or .01; Figure S10b). Conversely, fewer cDC2 and pDC were observed in the spleen following immunization with CFA (Figure 7a). Importantly, we found that the expression of major histocompatibility complex class II molecule (MHC‐II) on DCs and macrophages was significantly lower in mice immunized with CFA compared to those receiving TLR ligands (Figure 7b and Figure S10c). This suggests a deficiency in antigen presentation function in the CFA‐immunized mice. Furthermore, we detected higher levels of IL‐10 in mice immunized with CFA, as measured by ELISA (Figure 3c) and flow cytometry (Figure S11a). Next, we sought to determine the cellular source of IL‐10 and found that neutrophils produced more IL‐10 than other innate immune cells (mast cell,^36^ monocyte,^37^, ^38^ macrophage, neutrophil, DC as gated in Figure S11b) or lymphocytes (NK cell, B cell, Tfh, Tfr, Treg and CD8^+^ T cell) (Figure 7c and Figure S11c). To analyse the cellular source of IL‐10, we gated on total IL‐10^+^ cell and further characterize the cell composition (Figure S11b). We found the main cell population of IL‐10^+^ cell include macrophage, neutrophil, B cell, among them neutrophils comprise 20−60% of total IL‐10^+^ cell both in OVA and rDsg3 immunization experiment (Figure S11d), indicating they might be the predominant source. Additionally, CFA immunization resulted in a higher proportion of IL‐10‐producing neutrophil (*p *< .01; Figure 7d and Figure S11e), accounting for approximately half of the total IL‐10^+^ cells. The lower level of TNF‐α^+^:IL‐10^+^ ratio induced by CFA+rDsg3 also indicated the neutrophil might be an anti‐inflammation profile (Figure S11e). This increased production of IL‐10 by neutrophils contributed to the dampening of immune responses. In conclusion, TLR ligands appeared to promote DC maturation and accumulation more effectively than CFA. Furthermore, they reduced the proportion of IL‐10‐producing neutrophils. These effects collectively shaped the specific CD4^+^ T cell responses in the experimental system.

## 3 DISCUSSION

Our study conducted a comprehensive comparison of the adaptive and innate immune responses to self and non‐self‐antigens induced by adjuvanted proteins. We provided evidence to suggest that TLR ligands possess favourable properties in the mouse model of PV. Additionally, we successfully developed an active mouse model of PV that eliminates the requirement for either Dsg3 knockout mice or immune‐deficient mice.^10^, ^11^ This model holds great potential for advancing the development of antigen‐specific therapies for PV, facilitating research and therapeutic interventions in this field. Our findings contribute to a deeper understanding of immune responses and offer valuable insights for the design and optimization of adjuvant strategies in the context of autoimmune diseases such as PV.

Immunization with self‐antigen is a commonly used and effective method for inducing autoimmune diseases in animal models. Examples include myelin oligodendrocyte glycoprotein (MOG) in multiple sclerosis,^5^ collagen in rheumatoid arthritis,^39^, ^40^ retinal antigen in experimental autoimmune uveitis^41^ and insulin in type 1 diabetes.^42^ This approach elicits immune responses specific to the autoantigen, making it suitable for studying the development of targeted therapies or antigen‐specific treatments. Previous studies demonstrated that antibodies induced by immunization with rDsg3 in wild‐type mice did not recognize the native Dsg3 protein.^11^ The adoptive transfer of *Dsg3^−/−^* lymphocytes into *Rag2^−/−^* mice results in the induction of stable anti‐Dsg3 IgG production, accompanied by the development of PV.^11^ Other researchers found that transferring T cells from rDsg3‐immunized wild‐type mice to T cell‐deficient mice could promote the differentiation of recipient naïve B cells and the production of anti‐Dsg3 IgG, as assessed by rDsg3‐coated ELISA. However, they did not test the pathogenicity of the serum from recipient mice, including the presence of antibodies recognizing native Dsg3.^19^ Notably, the adjuvants used in these studies were CFA, alum,^15^, ^19^ TiterMax^18^ or unspecified.^11^ We believe that the recent availability of vaccine adjuvants with excellent immune‐activation capabilities opens up possibilities for inducing Dsg3‐specific autoimmune responses. The immunostimulatory effects of TLR ligands have been extensively documented,^43^, ^44^, ^45^, ^46^, ^47^, ^48^ therefore, we investigated the potential of TLR ligands to disrupt self‐tolerance against Dsg3. To the best of our knowledge, this is the first report demonstrating that a PV‐like phenotype can be directly induced in wild‐type mice through immunization with rDsg3 and TLR ligands. The PV phenotype closely resembles that of the adoptive transfer model, exhibiting similar gross, histological and serological characteristics. The underlying mechanism may involve impaired regulatory T cells, but the specific pathways responsible will be further investigated in future studies. We anticipate that this method could also be applied to animal models of other autoimmune disease with identified autoantigens, especially autoimmune bullous diseases, such as bullous pemphigoid, whose autoantigen was found to be BP180.^49^

The presence of anti‐Dsg3 IgG detected in mice that received rDsg3 and CFA without exhibiting a PV phenotype could be attributed to two factors. Firstly, the rDsg3 used for immunization was constructed with a histidine tag. Consequently, the immunization might have led to the production of anti‐histidine antibodies. Secondly, immunization with a protein antigen like rDsg3 can generate a diverse repertoire of B cells, targeting different epitopes along the Dsg3 protein.^50^, ^51^, ^52^, ^53^ As a result, some of the antibodies produced by those B cells may not recognize the native form of Dsg3, implying that they are not pathogenic in nature. It was demonstrated that Abl family tyrosine kinases inhibitor reduced circulating IgG extravasation in the skin and attenuated the murine pemphigus,^54^ whether CFA prevented the development of PV via impairing IgG extravasation could be explored in the future.

It is worth noting that some mice did not develop PV symptoms after immunization in wild‐type mice. We observed a higher incidence in experiments conducted during winter, with nearly all mice receiving rDsg3 and TLR ligands developing PV symptoms, whereas mice without symptom were primarily observed in experiments conducted during summer. From our point of view, the heated air of animal facility in winter might play a role in the pathogenesis of PV as it was reported that increasing temperatures are associated with increased hospitalization for pemphigus.^55^ Discussions on PV pathogenesis have also explored associations with seasons or temperatures,^56^, ^57^ with studies from South Africa observed exacerbation of pemphigus during summer time,^58^ and conflicting findings from Iran showed that higher rate of disease onset was in winter.^59^ On the other hand, the outcome of CFA is not related with the temperature. Our novel model could, therefore, serve to investigate environmental factors affecting PV.

We further optimized the adoptive transfer method by replacing CFA with TLR ligands, which results in a robust PV phenotype characterized by body weight loss, skin erosion and the formation of intra‐epithelial blisters. From our perspective, the adoptive transfer PV model relies on the use of two species of gene knockout mice, and the high costs associated with these mice may limit its widespread application. In contrast, direct immunization in wild‐type mice could be a more cost‐effective and accessible approach for advancing research in this field. Another notable difference between these two animal models of PV is the acquisition and loss of self‐tolerance. In the adoptive transfer model, recipient mice lack immune tolerance to any antigen due to the absence of mature T and B cells. In contrast, active immunization is based on the breakdown of pre‐established self‐tolerance to Dsg3, closely resembling the clinical pathogenesis of PV. Furthermore, the magnitude of the immune response differed between the two models. In the adoptive transfer model, the activity of donor cells transferred into immune‐deficient recipient mice is not fully restricted or regulated. This means that donor cell responses are essentially unlimited and could theoretically be infinitely activated, potentially making them difficult to control using certain regulatory immunotherapies. On the other hand, direct immunization in wild‐type mice can mimic the breach of tolerance that occurs during the physiological development of PV. In this scenario, the presence of pre‐existing regulatory cells may play a role in future therapeutic approaches.

In our study, we observed a notable difference between CFA and TLR ligands in terms of the presence of regulatory cells in draining LNs and spleen. This difference may be attributed to the increased IL‐10 produced by neutrophils, but further investigation is necessary to determine whether these neutrophils possess suppressive properties.^60^ Previous studies have demonstrated that Tfr cells are induced following immunization with incomplete Freund's adjuvant and CFA, mediated by PD‐L1.^61^ In our study, we discovered that chemokine CCL17 might contribute to the suppression of the overall CD4^+^ T cell response. It has been well stated that mature DCs preferentially attract Treg cells among circulating CD4^+^ T cells, by secretion of CCR4 ligands CCL17 and CCL22,^62^ and CCL17 and CCL22 chemokines within tumour microenvironment are related to the accumulation of Foxp3^+^ regulatory T cells in gastric cancer.^63^ This property has been applied in tumour therapy by targeting CCR4, the ligand of CCL17 to reduce Treg suppression.^34^, ^64^ While CCL17 has been associated with protective roles in myocardia disease,^65^, ^66^ studies in tumour immunology have found that CCL17‐mediated recruitment of Tregs promotes tumour progression and resistance.^67^ Patients with lower levels of CCL2^+^ or CCL17^+^ cells in their tumours have shown longer survival times compared to those with higher numbers of these cells.^67^ In addition, the antigenic properties of the immunogen may also influence Treg induction. For instance, GMCSF‐MOG emulsified in CFA led to a higher proportion of Tregs than MOG clone.^68^ Therefore, targeting Treg migration could be a promising approach for the development of novel therapies.^39^, ^69^

Our study has some limitations that should be acknowledged. Firstly, our focus was primarily on TLR ligands, specifically TLR3, TLR4 and TLR9, as representative active adjuvants. While we did test other adjuvants such as AddaVax (similar to MF59 in influenza vaccine) and alum,^28^, ^70^ a more comprehensive comparison should encompass additional adjuvant candidates to thoroughly assess their properties and effects. Secondly, in our analysis, we gated on total B cells and did not specifically examine antigen‐specific cells. This approach includes bystander cells and limits the precision of information regarding the effects of adjuvants.^5^, ^61^, ^71^, ^72^, ^73^

## 4 CONCLUSIONS

**FIGURE 8:**
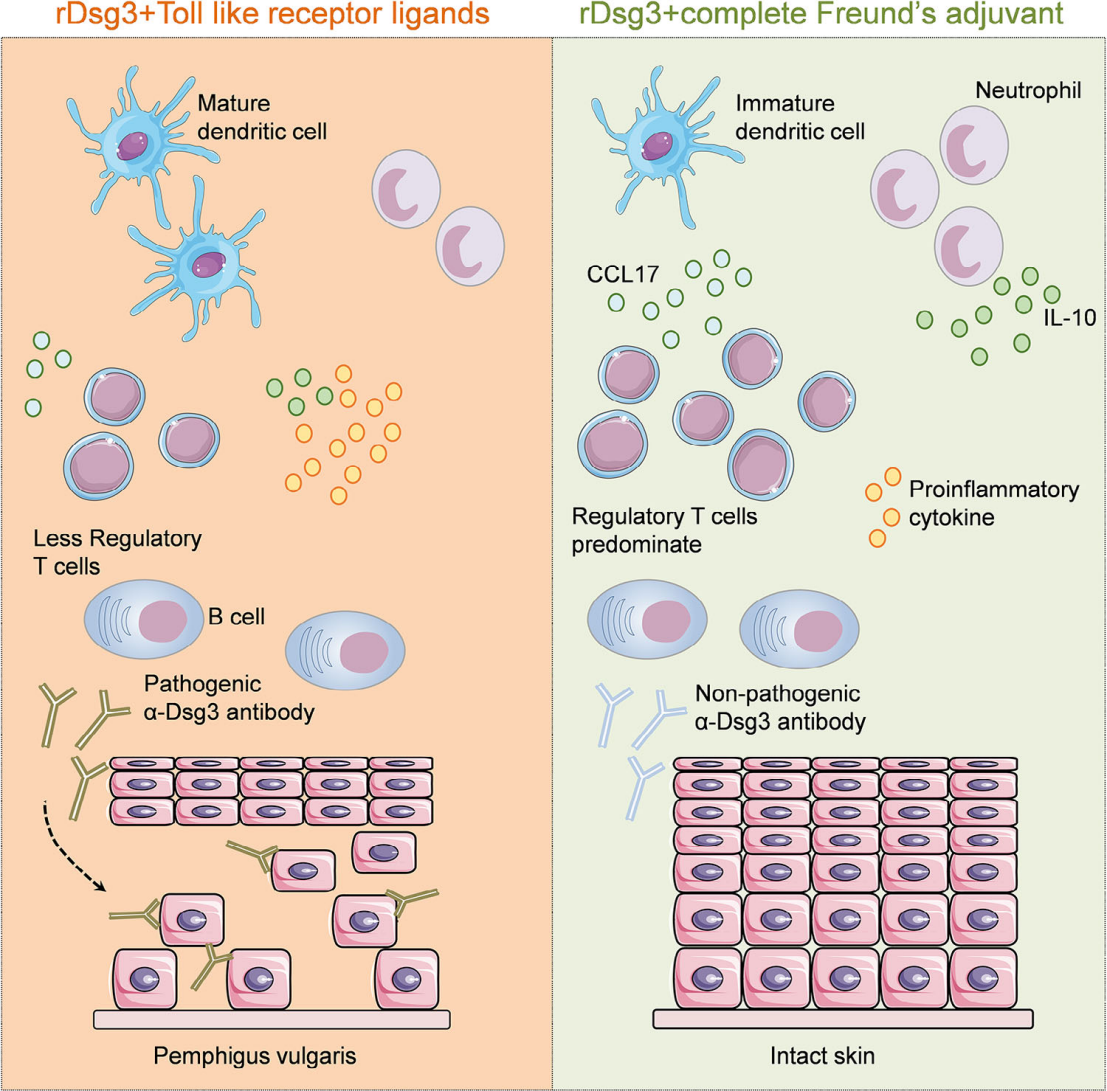
Immunostimulatory effect of TLR ligands and CFA in mouse model of pemphigus vulgaris.

In conclusion, our study demonstrates the potential of TLR ligands in breaking self‐tolerance and inducing a PV‐like phenotype in wild‐type mice through immunization with rDsg3 (Figure 8). This active mouse model of PV offers advantages over the adoptive transfer model in terms of closer resemblance to the clinical pathogenesis of the disease. The differential effects of adjuvants on the immune response and the involvement of regulatory cells highlight the importance of selecting appropriate adjuvants for reshaping autoimmune responses. Further research is needed to explore additional adjuvants, investigate the mechanisms underlying immune tolerance and activation, and identify potential therapeutic targets. These findings contribute to our understanding of immune responses and the optimization of adjuvant strategies in the context of autoimmune diseases such as PV.

## 5 METHODS

### 5.1 Animals and reagents

C57BL/6J mice (neonatal or 6−8 weeks old) were obtained from GemPharmatech or Vital River. C57BL/6J *Dsg3^–/–^* mice were constructed by the Shanghai Model Organism Center. C57BL/6J *Rag2^–/–^* mice were provided by Cyagen or Huafukang. All mice were 6−8 weeks old, female, specific‐pathogen free, unless otherwise specified. Mice were randomly grouped and allowed to adopt for at least 7 days before experiments. To minimize potential confounders, the order of treatments and measurements, or animal/cage location were changed often. Animal experiments were approved by the Institute of Dermatology Animal Authorities.

Recombinant mouse Dsg3 protein (rDsg3) with a histidine‐tag was produced by DetaiBio. OVA was obtained from Sigma Aldrich. Poly(I:C) and MPLA were purchased from InvivoGen. CFA was obtained from Invitrogen. CpG1826 was synthesized by Sangon Biotech. Epitope peptides (ova_257‐264_ with amino acid sequence SIINFEKL; ova_323‐339_ with amino acid sequence ISQAVHAAHAEINEAGR) were synthesized by the Chinese Peptide Company.

### 5.2 Immunization

Five micrograms of protein was mixed with adjuvants and administered via subcutaneous injection at the footpad (f.p.) in a volume of 50 μL for 3 consecutive days (day 0, 1 and 2). The daily doses of adjuvants were as follows: 30 μg poly(I:C) + 3 μg CpG ODNs, 3 μg of MPLA and 25 μL of CFA. Mice were boosted on days 14, 15 and 16. Each group and time point of the experiments included a minimum of three animals or samples. Unimmunized mice were used as an untreated control group.

### 5.3 Passive transfer neonatal pemphigus model

Neonatal C57BL/6J mice (approximately 24 h after birth) were intradermally injected with anti‐Dsg3 antibodies (clone AK23^54^, ^74^, ^75^ from MBL, or 5G11^76^ from Santa Cruz), along with .1 μg of exfoliative toxin A (ETA, from Toxin Technology Inc.), in a volume of 50 μL. After 16–24 h, the mice were clinically evaluated by a blinded and experienced dermatologist, who applied mechanical stress to the back and sides of the mice to observe the Nikolsky phenomenon. Skin tissues were then harvested for histological analysis.

### 5.4 Adoptive transfer pemphigus model

To establish the adoptive transfer model of PV, *Dsg3^–/–^* mice were verified by PCR and/or western blot analysis. These female mice were then immunized with rDsg3 and active adjuvants through subcutaneous injection at the footpad (f.p.), using a volume of 50 μL on days 0, 1 and 2. A booster was administered on days 14, 15 and 16. At either 35 or 49 days after the initial immunization, lymphocytes were isolated from both naïve or immunized *Dsg3^–/–^* mice. These lymphocytes were transferred to sex‐matched *Rag2^–/–^* mice via intravenous injection through the tail vein, using the indicated number of cells being used.

### 5.5 Flow cytometry

Cells from LNs or spleen were isolated at the indicated times after immunization. Antibodies for flow cytometry were purchased from BioLegend or eBiosciences, unless otherwise noted. MHC‐I tetramers (tet) loaded with ova_257‐264_ peptides (H2K^b^‐ ova_257‐264_ tetramer, referred as tet257) and MHC‐II tetramers loaded with ova_329‐337_ peptides (IA^b^‐ ova_329‐337_ tetramer) were obtained from the NIH Tetramer Core Facility. For staining of MHC‐II tetramer, cells were incubated with peptide epitope for 8 days before surface staining. Cells were incubated with antibodies against various cellular markers. To measure intracellular cytokines in T cells, cells were stimulated for 5 h at 37°C with peptide at 100 nM or with PMA and ionomycin in the presence of 1 μg/mL of brefeldin A. Anti‐CD107a (clone eBio1D4B) mAbs were added along with the peptide upon restimulation. For intracellular cytokine staining, cells were fixed and permeabilized and then incubated with antibodies against intracellular cytokines. For staining of intranuclear transcription factors (Ki‐67, Foxp3), cells were processed using a staining kit (eBiosciences) following the manufacturer's instructions.

Sample data were acquired using a FACSVerse (BD) or Northern Light‐CLC Aurora full‐spectrum flow cytometer (Cytek) and analysed with FlowJo software (TreeStar Inc).

### 5.6 Antigen‐specific antibody measurement

Serum or plasma was collected on the indicated days after the first immunization. OVA‐ or Dsg3‐specific antibodies were measured by ELISA as previously described.^77^ Briefly, ELISA plates (Nunc) were pre‐coated with antigen protein at 5 μg/mL overnight and then blocked with BSA containing PBS for 2–15 h. Sera or plasma were serially diluted according to the previous report^77^ and incubated in ELISA plates for 2 h. The plates were incubated with HRP‐conjugated anti‐mouse IgG, IgG1, IgG2c or IgG3 detection antibodies (Southern Biotechnology Associates) for 2 h. Colour development was achieved by incubating with the TMB substrate solution for 5–30 min in darkness. The OD was read at 450 nm using a plate reader (Biotek).

### 5.7 Cytokine measurements

To determine cytokine levels following immunization, the draining LN was removed and homogenized. After centrifugation, CCL17 and IL‐10 levels in the supernatants were measured by ELISA, following the manufacturer's protocols (CSB‐E04594m and CSB‐E14144m, Cusabio).

### 5.8 PV score and histological analysis

The PV score was assessed by a blinded experienced dermatologist, based on the number of sites with skin erosion, alopecia and erythema as listed in Tables 1 and 2, as previously reported.^12^, ^13^ Only the first author (CXG) was aware of the group allocation at the different stages of the experiment (during the allocation, the conduct of the experiment, the outcome assessment and the data analysis). At indicated time points, skin tissue with erosion was dissected. Some of the tissue was snap‐frozen and stored for protein or mRNA measurement, while the rest was flattened on a piece of aluminium foil. Half of the flattened skin was used for frozen sectioning, and the other half was fixed in 4% paraformaldehyde (Biosharp) for 48 h. The fixed tissues were dehydrated, paraffin embedded, and 5 μm sections cut and dried. For histopathological analysis, the sections were deparaffinized and stained with haematoxylin/eosin (Solarbio), following the manufacturer's protocols. To determine IgG deposition, frozen sections were washed, dried and incubated with anti‐mouse IgG‐FITC (Abclonal). The binding of anti‐Dsg3 IgG was visualized using an Olympus BX53 microscope and analysed with cellSens (v3.1) software (Olympus) or Mantra Quantitative Pathology Workstation (Akoya Bioscience).

**TABLE 2:** Summary of pemphigus vulgaris score and the incidence of phenotypic symptoms in the adoptive transfer PV model.

			Incidence (%)			
Type of lesion	Sites	Score	Untreated	MPLA^a^	PC^a^	CFA^a^
Erosion	muzzle	1	0	60	67	0
	periocular	1	0	100	83	75
	periauricular	1	0	80	67	0
	back	1	0	0	0	25
	chest	1	0	0	17	0
	abdomen/anus	1	0	0	33	0
	right foreleg	1	0	40	83	0
	left foreleg	1	0	80	100	0
	right hind leg	1	0	40	50	0
	left hind leg	1	0	40	67	0
	tail	1−2	0	80	50	0
Alopecia	face	1	0	60	83	25
	neck	.5	0	80	83	50
	back	1	0	60	50	75
	abdomen	.5	0	20	17	75
Erythema	footpad	1	0	20	0	0
	abdomen	1	0	0	0	0

Abbreviation: PC, poly(I:C)+CpG 1826.

^a^ MPLA, PC and CFA represent different conditions in the experiment. Specifically, they refer to *Rag2^–/–^* mice receiving donor cells from *Dsg3^–/–^* mice that were previously immunized with rDsg3 and MPLA, PC or CFA, respectively.

### 5.9 Transcriptome and gene expression

RNA was isolated from frozen skin tissue using the TRIzol reagent (Invitrogen). After qualification by 5300 Bioanalyser (Agilent) and quantification by the ND‐2000 (NanoDrop Technologies), RNA purification, reverse transcription, library construction and bulk RNA‐sequencing were conducted according to the manufacturer's instructions (Illumina). The expression level of each transcript was calculated according to the transcripts per million reads to identify differential expression genes (DEGs) between groups. Genes with |log2FC|≥1 and FDR≤.05 or .001 were considered to be significantly different expressed genes. Functional‐enrichment analyses were performed to identify which DEGs were significantly enriched in gene ontology (GO) terms and metabolic pathways at Bonferroni‐corrected *p*‐value≤.05 compared with the whole‐transcriptome background. Data visualization was performed using the Majorbio online platform. The analysed data were uploaded as Table S1 in Supplementary Materials.

### 5.10 Statistical analysis

Comparison between the two groups was analysed by Student's *t*‐test, whereas comparisons among the means of more than two groups were determined by one‐way ANOVA post hoc analysis with Bonferroni correction. Data were visualized by GraphPad Prism. *p* Values less than .05 were considered statistically significant and denoted by *p < .05, **p < .01 and ***p < .001. No outliers were removed and all data were included for analysis.

## AUTHOR CONTRIBUTIONS

Changxing Gao, Zijun Wang and Qianjin Lu conceived the study. Changxing Gao performed most experiments, interpreted the data and wrote the manuscript. Mei Liu, Xinyu Fan, Yue Xin, Yong Zeng, Bo Zhang and Cheng Zhao participated in some experiments. HY conducted a western blot and participated in some experiments. Lingzhi Zhang and Jing J. Li performed the tetramer staining. Mei Liu, Ming Zhao, Zijun Wang and Qianjin Lu assessed and verified the reported data. Ming Zhao, Zijun Wang and Qianjin Lu revised the manuscript and oversaw the overall execution of the projects.

## CONFLICT OF INTEREST STATEMENT

The authors declare no conflict of interest exists.

## ETHICS STATEMENT

Animal studies and animal procedures in this study were conducted in accordance with animal welfare and approved by the Institute of Dermatology Animal Authorities.

## Supporting information

Supplemental Information

Supplemental Information

## Data Availability

All data will be available upon publication of the manuscript, by contacting the corresponding author.
