# Supplementary material for: Immunostimulatory effects of Toll‐like receptor ligands as adjuvants in establishing a novel mouse model for pemphigus vulgaris
Source: Clin Transl Med. 2024 Jul 19;14(7):e1765. doi: 10.1002/ctm2.1765 (PMC11259602; doi:10.1002/ctm2.1765)
Supplement: Supplementary file 1 — Supplemental Information [file CTM2-14-e1765-s001.docx]

## Supplemental information

### Immunostimulatory Effects of Toll-like Receptor Ligands as Adjuvants in Establishing a Novel Mouse Model for Pemphigus Vulgaris

Changxing Gao^1^, Mei Liu^1^, Yue Xin^1^, Yong Zeng^2^, Hui Yang^3^, Xinyu Fan^1^, Cheng Zhao^1^, Bo Zhang^1^, Lingzhi Zhang^4^, Jing J Li^4^, Ming Zhao^1*^, Zijun Wang^2,5*^, Qianjin Lu^1*^

^1^ Key Laboratory of Basic and Translational Research on Immune-Mediated Skin Diseases, Chinese Academy of Medical Sciences,

Jiangsu Key Laboratory of Molecular Biology for Skin Diseases and STIs,

Hospital for Skin Diseases, Institute of Dermatology, Chinese Academy of Medical Sciences and Peking Union Medical College, Nanjing, China

^2^ Department of Dermatology, The Second Xiangya Hospital of Central South

University, Changsha, China

^3^ Drum Tower Hospital Affiliated to Medical School of Nanjing University, Nanjing, China

^4^ State Key Laboratory of Bioactive Substance and Function of Natural Medicines, Beijing Key Laboratory of New Drug Mechanisms and Pharmacological Evaluation Study,

Department of Pharmacology, Institute of Materia Medica, Chinese Academy of Medical Sciences and Peking Union Medical College, Beijing, China

^5^ Laboratory of Molecular Immunology, The Rockefeller University, New York, NY, USA

^*^Correspondence to: Qianjin Lu (qianlu5860@pumcderm.cams.cn), Zijun Wang (okvinci@126.com), Ming Zhao (zhaoming307@126.com)

**This suppliment file includes:**

Fig. S1 to S11; caption of Table. S1

## Supplementary Figures


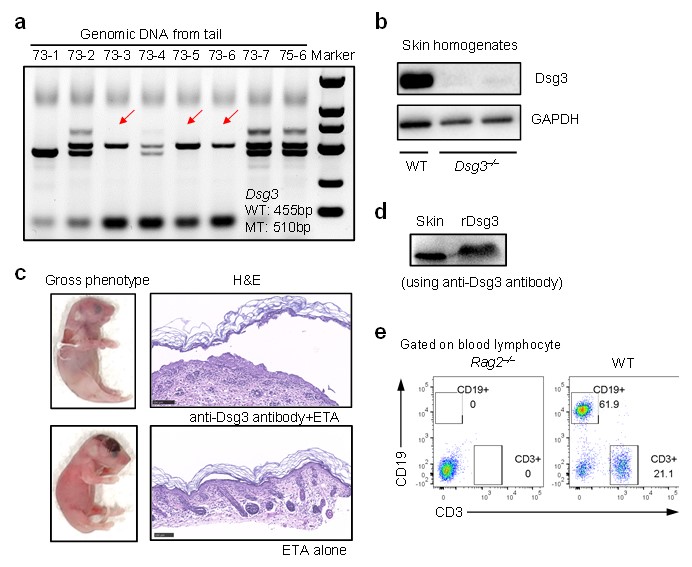


**Fig. S1. Material preparation and verification conducted in this study.** (**a**) *Dsg3^–/–^* mice were generated through targeted mutation. Genomic DNA was extracted from mouse tail samples, and homozygotes were identified by PCR. Red arrow indicates the band representing homozygotes, which appeared at approximately 510 bp. (**b**) Skin samples obtained from *Dsg3^–/–^* mice were homogenized for western blot analysis to confirm the absence of Dsg3. Skin samples from wild-type (WT) mice were used as positive control. (**c**) The specificity of the anti-Dsg3 antibody was validated by passive neonatal PV model. The Nikolsky phenomenon was examined, and skin tissues were harvested for histological analysis. (**d**) The antigenicity of rDsg3 was verified by western blot analysis by incubating with the validated anti-Dsg3 antibody. (**e**) *Rag2^–/–^* mice were routinely verified by flow cytometry. Lymphocyte was isolated from peripheral blood and underwent red cell lysis, surface antigen staining and analyzed by flow cytometry. Results are representative of at least two independent experiments.


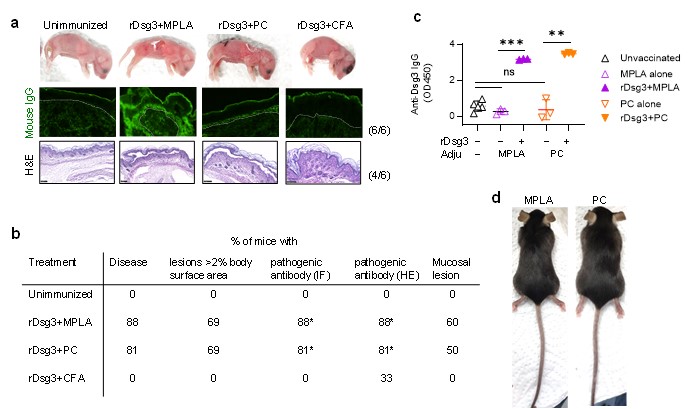


**Fig. S2. Characterization of antibody pathogenicity and disease phenotype of immunization-induced PV model.** (**a**) The pathogenicity of antibody was evaluated by the passive transfer neonatal pemphigus model. Neonatal C57BL/6J mice (approximately 24 hours after birth) were intradermally injected with serum from the immunization-induced pemphigus model (40 day after modeling), the Nikolsky phenomenon was examined. Skin tissues were then harvested for H&E and IF. “4/6” means there was no detachment by H&E examination in four out of six tested plasma; and “6/6” denoted no IgG deposition was detected by IF in all six tested plasma. (**b**) Summary of the incidence of phenotypic symptoms in the immunization-induced PV model. *, the percentage was an estimate because mice receiving rDsg3 and TLR ligands with no PV symptom were not evaluated for the presence of pathogenic antibody. Data were pooled from two experiments. (**c**) Dsg3-specific IgG was measured by ELISA. OD values at 1:100 dilution read at 450nm are shown. (**d**) The gross phenotype of mice receiving MPLA or PC alone.

**Fig. S3. A higher number of donor cells primed with CFA was required for the induction of PV.** *Dsg3^–/–^* mice were immunized with rDsg3 and various adjuvants following a prime-boost regimen. After 35 days of the initial immunization, lymphocytes were isolated from the spleen and injected into *Rag2^–/–^* mice with different cell numbers through the tail vein. The cell numbers are indicated as 1e7, representing 1×10^7^ cells. After 40 days of cell transfer, plasma or serum was prepared from peripheral blood, and Dsg3-specific IgG levels were measured by ELISA. OD values at 450nm are shown.

**Fig. S4. CD4^+^ T cell responses induced by OVA and TLR ligands.** Mice were immunized with OVA and different adjuvants. Draining lymph nodes were harvested for flow cytometry analysis at 7 days after the first immunization. The cell number and proportion of total CD4^+^ T cells (**a**), Tfr and Tfh cells (**b**) in pLN (upper panel) and spleen (lower panel) were compared between different adjuvants. (**c**) Flow cytometry analysis of Tfh and Tfr cells in pLN: CD8^–^CD4^+^CXCR5^+^PD-1^+^ T cells were gated for the analysis of the proportion of Tfh and Tfr in pLN. (**d**) Flow cytometry analysis of Foxp3^+^ CD4^+^ Tregs in the draining lymph node, gated on CD8^–^CD4^+^ T cells. The results are representative of two independent experiments and shown as mean ± SD. Statistical significance between groups is indicated by **p*<0·05 and ***p*<0·01.

**Fig. S5. Immunization with rDsg3 and TLR ligands induced higher CD4^+^ T cell responses than CFA.** (**a**) A schematic depicting the treatment of mice is shown. Mice were immunized with rDsg3 and different adjuvants in a prime-boost regime as in **Fig.1b**. Spleens were harvested for flow cytometry analysis 35 days after the first immunization. Treg (**b**) and Tfr (**c**) in the spleen were gated for analysis of the expression of activation markers. (**d**) Flow cytometry analysis of CD4^+^ T cell memory phenotyping. (**e**) Flow cytometry analysis of CD4^+^ T cell functionality by intracellular cytokine staining. The results are representative of two independent experiments and shown as mean ± SD. **p*<0·05 indicate a significant difference between groups. MFI, mean fluorescence intensity; Tcm, Central Memory T cell; Tem, Effector Memory T cell, and ns represents not significant.

**Fig. S6. Antigen specific CD4^+^ T cell responses.** (**a**) Mice were immunized with OVA and various adjuvants following a prime-boost regime. Spleen samples were collected 35 days after the prime immunization and stimulated with peptide before tetramer staining. (**b**) Gating strategy for the analysis of Tet^+^CD4^+^ T cells. Total live cells were gated for analysis. (**c**) The proportion of Treg, Tfr and memory cell subsets were analyzed by flow cytometry. The presented results are representative of two independent experiments and shown as mean ± SD. Statistical significance between groups is indicated by **p*<0·05 and ***p*<0·01.

**Fig. S7. Adjuvants induced similar primary antigen-specific CD8^+^ T cell responses.** Mice were immunized with OVA and indicated different adjuvants as in **Fig.S4**. Draining lymph nodes were harvested for flow cytometry analysis 7 days after the first immunization. (**a,b**) The frequency and cell number of total CD8^+^ T cells (**a**) and antigen-specific CD8^+^ T cells (**b**). (**c**) Flow cytometry analysis of Tet^+^CD8^+^ T cell functionality by CD107a and intracellular cytokine staining. (**d**) Tet^+^CD8^+^ T cells were gated for the analysis of the proliferation capacity and activation status of antigen-specific CD8^+^ T cells by examining the expression of Ki-67 and CD44. The presented results are representative of two independent experiments and shown as mean ± SD. Statistical significance between groups is indicated by **p*<0·05 and ***p*<0·01.

**Fig. S8. TLR ligand and rDsg3 produced comparable CD8^+^ T cell and B cell responses.** (**a**) The activation status of CD8^+^ T cells were examined by the expression of Ki-67 and CD44. (**b**) Flow cytometry analysis of CD8^+^ T cell phenotyping. (**c**) The proportion and absolute number of B cell subsets were analyzed by flow cytometry. The presented results are representative of two independent experiments and shown as mean ± SD. Statistical significance between groups is indicated by **p*<0·05. GC denotes germinal center, and ns represents not significant.

**Fig. S9. Gating strategy for analysis of B cell subsets by flow cytometry.** (**a**) Gating strategy for analysis of B cell subsets by flow cytometry. (**b**) Mice were immunized with OVA and indicated different adjuvants as in **Fig.S4**.Draining lymph nodes were harvested for flow cytometry analysis 7 days after the first immunization. The frequency and cell number of B cell subsets cells was shown. The presented results are representative of two independent experiments and shown as mean ± SD. Statistical significance between groups is indicated by **p*<0·05, ***p*<0·01 and ****p*<0·001.

**Fig. S10. CFA did not induce fully activation of antigen presenting cells during priming phase.** (**a**) Gating strategy for analysis of granulocyte cell subsets by flow cytometry. Mice were immunized with OVA and indicated different adjuvants as in **Fig.S4**. Draining lymph nodes were harvested for flow cytometry analysis 7 days after the first immunization. (**b**) The frequency and cell number of DC cell subsets and macrophage in the pLN. (**c**) The presentation functionality of cDC2 and macrophage in pLN were characterized by the expression of MHC-II. The presented results are representative of two independent experiments and shown as mean ± SD. Statistical significance between groups is indicated by **p*<0·05, ***p*<0·01 and ****p*<0·001.

**Fig. S11. Neutrophil were the major source of elevated IL-10 in mice** **immunized with CFA.** (**a**) The frequency of IL-10^+^ cell in total live splenocytes in mice immunized with OVA. (**b**)The gating strategy for the analysis of Mast cell, monocyte, DC subsets, macrophages, neutrophil, lymphocyte (NK cell, B cell, CD4^+^T subsets, CD8^+^T cell and some undefined cells) by flow cytometry is shown. (**c**) Comparison of the capacity of IL-10 production of major innate immune cells and lymphocytes. (**d**)The frequency of different subsets in IL-10^+^ cells in mice immunized with rDsg3 (upper panel) or OVA (lower panel). (**e**) The frequency of IL-10^+^ /Neutrophil, TNF-α^+^/Neutrophil, TNF-α^+^/ IL-10^+^ in mice immunized with rDsg3. The presented results are representative of two independent experiments and shown as mean ± SD. Statistical significance between groups is indicated by **p*<0·05 and ***p*<0·01.

**Table. S1.** The analyzed data of RNA-seq.
